# Zebrafish Syndromic Albinism Models as Tools for Understanding and Treating Pigment Cell Disease in Humans

**DOI:** 10.3390/cancers14071752

**Published:** 2022-03-30

**Authors:** Sam J. Neuffer, Cynthia D. Cooper

**Affiliations:** School of Molecular Biosciences, Washington State University Vancouver, Vancouver, WA 98686, USA; samantha.neuffer@wsu.edu

**Keywords:** melanocytes, melanin synthesis, Hermansky–Pudlak Syndrome, albinism, zebrafish

## Abstract

**Simple Summary:**

Zebrafish (*Danio rerio*) is an emerging model for studying many diseases, including disorders originating in black pigment cells, melanocytes. In this review of the melanocyte literature, we discuss the current knowledge of melanocyte biology relevant to understanding different forms of albinism and the potential of the zebrafish model system for finding novel mechanisms and treatments.

**Abstract:**

Melanin is the pigment that protects DNA from ultraviolet (UV) damage by absorbing excess energy. Melanin is produced in a process called melanogenesis. When melanogenesis is altered, diseases such as albinism result. Albinism can result in an increased skin cancer risk. Conversely, black pigment cell (melanocyte) development pathways can be misregulated, causing excessive melanocyte growth that leads to melanoma (cancer of melanocytes). Zebrafish is an emerging model organism used to study pigment disorders due to their high fecundity, visible melanin development in melanophores (melanocytes in mammals) from 24 h post-fertilization, and conserved melanogenesis pathways. Here, we reviewed the conserved developmental pathways in zebrafish melanophores and mammalian melanocytes. Additionally, we summarized the progress made in understanding pigment cell disease and evidence supporting the strong potential for using zebrafish to find novel treatment options for albinism.

## 1. Introduction

Melanin is a category of pigments that range in color from yellow-red (pheomelanin) to brown and black (eumelanin), and they are produced by enzymes in a process called melanogenesis [1]. The diverse variety of organisms that produce melanin are adapted to reduce the consequences of ultraviolet (UV) radiation and DNA damage, including altered development, cancer, skin damage, immunosuppression, increased mortality, and reduced quality of life [2,3,4,5]. In animals exposed to UV radiation from the sun, melanin protects DNA from mutations by absorbing UV light and free radicals [6]. After UV exposure, additional skin cells (keratinocytes) respond to UV stress by synthesizing and secreting the melanocortin stimulating hormone (MSH) [7,8]. MSH then interacts with melanocortin receptor 1 (MCR1) on the surface of melanocytes (melanin producing cells) to activate a signaling cascade that directs melanocyte specific organelles, melanosomes, to synthesize, store, and transport melanin within pigment cells [9]. Additional signaling pathways, including the endothelin and kit signaling pathways, are involved in promoting melanogenesis and normal melanocyte function. As a result of these signals, the increase in melanin is responsible for tanning and UV protection in humans [8].

Melanogenesis occurs in melanosomes, members of a category of lysosome-related organelles (LROs). LROs are a group of cell specific specialized trafficking and/or secretory compartments that use similar but incompletely understood mechanisms for their synthesis and function ([10] and more recently reviewed by Delevoye, Marks, and Raposo [11]). Relatedly, defects in proteins important for melanogenesis enzyme transport are correlated with transport defects in other LROs. For example, cytotoxic T lymphocytes generate LROs, lytic granules, using the similar intracellular transportation systems that melanocytes use to traffic melanin [12,13]. The content in lytic granules kills bacteria and cancerous cells [14,15]. When lytic granule generation is perturbed, Hermansky-Pudlak Syndrome (HPS; a form of albinism) patients are more susceptible to infection [13]. Without understanding the genetics of albinism, developing treatments for albinism may be inappropriately targeted to treating albinism symptoms without targeting the immune system malfunctions. Animal models, including zebrafish, are an excellent resource for developing specific treatment options for pigmentation disorders, including melanoma and albinism, due to the ability to characterize conserved melanogenesis and melanogenesis enzyme trafficking pathways [13]. This review will consider conserved mechanisms underlying pigment biology in mammals and zebrafish (*Danio rerio*), while highlighting gaps in knowledge regarding albinism biology. Additionally, we will discuss how zebrafish can fill those gaps and be used to better understand and treat albinism.

## 2. Advantages of Using Zebrafish to Study Albinism

House mice (*Mus musculus*) are traditionally used to study pigment diseases such as albinism because mice are mammals and share many of the genes and processes involved in pigment cell development pathways and melanin synthesis pathways misregulated in human albinism [16,17]. However, zebrafish offer some advantages to studying pigment development and how it is altered in albinism. First, many of the genes that control pigment development and melanocyte function in humans and mice are conserved in zebrafish as well as other chordates [18,19,20]. Some examples of conserved melanocyte genes controlling the analogous zebrafish cell type, melanophores, include microphthalmia-associated transcription factor (indicated as MITF in humans, Mitf in mice, and *mitf* in zebrafish), dopachrome tautomerase (DCT), tyrosinase (TYR), tyrosinase-related protein 1 (TYRP1), and oculocutaneous albinism 2 (OCA2) [21,22]. Because melanophores are present in see through skin (in larvae and in *casper* adults [23]), melanophore development can be observed in live samples in real time. Relatedly, several zebrafish mutants with defects in genes that regulate the master melanophore specification gene, *mitfa*, have been isolated, uncovering new regulatory roles for *foxd3*, *tfap2a*, and *colgate/hdac1* during melanophore specification from neural crest cells [24,25,26,27,28]. Additionally, zebrafish mutants with defects in melanophore differentiation and survival are clarifying novel roles for cathepsin protease, non-coding RNAs, and adhesion protein Jam3 during later stages of melanophore development [29,30,31]. Thus, zebrafish mutants are already providing insight to melanophore/melanocyte development mechanisms.

Second, zebrafish have a higher fecundity than mice. Pregnant female mice are sacrificed to collect approximately 6–10 embryos per mouse to study early pigment development, while zebrafish parents can be returned to their housing after collecting up to 200 eggs from a single spawning pair every 10 days [32,33,34,35]. Therefore, zebrafish larvae are highly amendable to large scale drug treatments. This approach can be used with zebrafish disease models to quickly find novel treatment options for albinism. In addition, genetic modification experiments to test the genetic mechanisms underlying pigmentation diseases can be quickly performed on more individuals.

Third, zebrafish are already providing mechanistic insight into albinism biology. Reduction of zebrafish biogenesis of lysosome-related organelles complex 1 (BLOC1) S5 gene (using morpholino knockdown techniques) recapitulates human syndromic albinism, Hermansky-Pudlak Syndrome (HPS) 11 phenotypes including a reduction in eye pigmentation, and cardiovascular defects. Signaling pathways, altered as a consequence of BLOC1S5 reduction, included delta/notch and vascular integrity signaling [36]. HPS genes 1, 3, 4, and 5 zebrafish models present with kidney tissue impairment and illustrate a previously underappreciated impact of HPS gene loss of function–renal disease [37]. Additionally, several genomic mutants (discussed below) with HPS phenotypes are available to further our understanding of HPS models and to find novel specific treatments using the advantages of the zebrafish model.

## 3. Mechanisms Underlying Zebrafish and Mammalian Pigment Cell Biology

### 3.1. Black Pigment Cell Anatomy and Function

Melanocytes and melanophores are both specialized cells that produce melanin. In mammals, melanocytes reside in the basal layer of the epidermis [38]. In fish, melanophores are located in both the epidermis and the hypodermis on top of muscle [39]. Melanocytes/melanophores consist of a central cell body and dendrites that project outwards. Melanosomes traffic pigment throughout the cell in both melanocytes and melanophores, and they are transferred to neighboring cells in human skin. Though still under debate as to how melanosome transfer to surrounding skin cells occurs, progress has been made thanks to new technologies and approaches for understanding this process [40]. As a result, several models for melanosome transfer have been proposed, including exo/phagocytosis and membrane fusion [41]. In contrast, melanophores retain their pigment within the melanophore [39]. Because zebrafish melanophore biology is very similar to mammal melanocyte biology, we intertwine discussion of developmental mechanisms in both cell types below.

Melanogenesis is the production of melanin by melanin-producing cells and a reduction in melanogenesis underlies all forms of albinism [1]. Melanogenesis requires the expression of genes that code for the proteins that directly synthesize melanin, as well as the genes necessary for trafficking pigment-producing enzymes into the melanosomes and promoting the correct function of those enzymes after their arrival [42,43,44]. Failure in any of these processes can cause pigment loss resulting in albinism. However, the specific mechanisms working to promote healthy LROs and to prevent albinism symptoms are poorly understood.

There are multiple types of melanin, but eumelanin is the primary pigment altered in albinism [45]. Eumelanin is a polymer that consists of repeating units of 5,6-dihydroxyindole (DHI) and 5,6-dihydroxyindole-2-carboxylic acid (DHICA) [1]. It strongly absorbs UV light, which gives melanin its photoprotective properties [46]. Eumelanin is synthesized in stages by one to three different enzymes. Tyrosine is converted into dopaquinone by tyrosinase. Dopaquinone is spontaneously converted into dopachrome, from which point it can be converted into eumelanin by tyrosinase (TYR) after losing the carboxyl group (COO), or it can be converted into DHICA by dopachrome tautomerase (DCT) and then into eumelanin by tyrosinase-related protein 1 (TYRP1) [47].

### 3.2. Regulation of Melanogenesis—Some Remaining Questions

The primary point of the transcriptional control of melanogenesis is MITF. As a transcription factor, MITF controls the expression of tyrosinase, dopachrome tautomerase, and tyrosinase-related protein 1—enzymes critical to the production of melanin in melanosomes. MITF is regulated via several pathways, some of which include: (1) the MC1R (melanocortin 1 receptor) pathway, (2) the wingless/integrated (WNT) pathway, (3) the c-KIT pathway, and (4) the endothelin B receptor (ETBR) pathway. Together, these conserved, well-studied signaling pathways regulate the development of melanin-producing cells in both mammalian and zebrafish skin [48,49,50,51,52,53,54,55,56,57,58,59,60,61,62,63,64,65].

Conversely, the processes that control the transport of melanogenesis enzymes and the trafficking of melanosomes are incompletely understood (recently reviewed in Ohbayashi and Fukuda, 2020). Melanosomes are trafficked along microtubule and actin filaments [66]. Melanosomes mature in four stages. Stage one consists of premelanosomes, which lack pigment. Striations form inside the melanosome at stage two to support the deposition of melanin produced in stage three, when melanin synthesis proteins, such as TYRP1, DCT, and TYR, are trafficked to the melanosome. The melanosome becomes darkly pigmented during stage four, when they are considered mature [66,67]. The protein, PMEL, promotes the formation of striations within stage one of maturing melanosomes. The striations are responsible for the oblong shape of the melanosome and provide space for the deposition of pigment; however, PMEL is not absolutely required for pigment formation [67,68,69]. PMEL must be proteolytically cleaved to form fibrils. HPS1 and HPS4 mutant cells do not efficiently cleave PMEL, suggesting that they are required for the efficient processing of PMEL [70].

The melanogenesis enzymes TYRP1, DCT, and TYR are all synthesized in the endoplasmic reticulum (ER) and are sorted from the trans-Golgi network. TYR and TYRP1 are trafficked to the early endosome, while DCT is trafficked via the secretory pathway from the Golgi directly towards the melanosome [66]. TYRP1 requires the biogenesis of lysosome-related organelle complex 1 (BLOC1) and BLOC2 to sort to melanosomes [71,72]. BLOC1 is a multi-subunit complex that includes the protein gene products of Pallidin, Muted, Cappucino, Dysbindin, Snapin, Blos1, Blos2, and Blos3 [73]. It works with KIF13A and actin to elongate endosomal tubules for the transport of TYRP1, while BLOC2 works to target TYRP1 to the stage two melanosome [66,72,74]. BLOC2 is also a multi-subunit complex composed of proteins coded by HPS3, HPS5, and HPS6 genes [66]. Different mouse disease models for many of the subunits in both BLOC1 and BLOC2 exist ([75,76,77,78,79,80], while there is limited availability of zebrafish models for the same subunits. Snow white is the only currently available HPS zebrafish model with defects in BLOC2. Snow white is a substitution mutation in HPS5, enabling researchers to study the biology of this novel HPS-causing mutation when the protein is expressed [81].

In a separate, incompletely understood pathway, the adaptor protein 3 (AP3) complex interacts with the cytoplasmic tail of tyrosinase to pinch off some of the early endosome (vesicle budding) and traffic tyrosinase to a stage two melanosome [66,82,83,84]. The BLOC1 complex interacts with AP3 to traffic tyrosinase, though this is not required [71,72,77]. The adaptor protein 1 (AP1) complex can also sort tyrosinase, though it does so weakly and only when AP3 cannot [84,85,86]. Therefore, the delivery of tyrosinase to the melanosomes can occur through functionally-redundant pathways [84]. Fusion of the AP3/tyrosinase vesicles to target organelles may be mediated by the HOPS complex. In yeast, the expression of Vps41 on mitochondrial membranes redirected AP3 to the mitochondria [87]. Mutations in *vps41* CRISPR knockouts cause pigmentation and neurological defects [88]. Mutations in the AP3 complex, such as in the cases of HPS2 and HPS10, result in syndromic albinism [13,89]. Notably, people with Hermanksy–Pudlak Syndrome type 10 have symptoms of hypotonia (decreased muscle tone), which is also present in people with mutations in VPS41 [13]. Whether AP3, VPS41, and/or BLOC1 work collaboratively to promote tyrosinase sorting is unclear.

The mechanisms for trafficking DCT are even less understood. DCT is trafficked from the Golgi to the stage two melanosome. Rab6 and ELKS facilitate the docking of Dct to the melanosome [66,90]. Dct, as well as TYR and TYRP1, requires Rab32/Rab38 for trafficking [91]. Rab32/Rab38 are proteins that exchange GDP for GTP, and they are called small GTPases. Rabs are involved in the specific targeting of protein cargo through interactions with other trafficking proteins such as BLOCs or motor proteins required for trafficking melanosomes along microtubules [66]. Chocolate mice (Rab38) are models for HPS due to defects in lung surfactant secretion [92,93], but no specific type of HPS in humans or zebrafish is associated with Rab38.

Once melanogenesis enzymes are in the melanosome, they can produce melanin. However, these enzymes require the proper environment to function correctly. Tyrosinase, tyrosinase-related protein 1, and dopachrome tautomerase require a specific pH inside the melanosome to allow the binding of copper, iron, and zinc cofactors, respectively [94,95]. The pH of the melanosome is regulated by proton pumps [91]. Some of the proteins thought to regulate melanosomal pH are MATP and OCA2, and mutations in these genes are associated with albinism [21,96,97]. Treatment of *oca2* mutant zebrafish with vacuolar ATPase (V-ATPase) inhibitor, bafilomycin 1A, rescues melanin synthesis, suggesting that melanosomal pH is regulated by V-ATPase [21]. An additional regulator of melanosome pH is the Oca2 protein itself. Acting as an anion channel in melanosomes, Oca2 may also regulate melanosome pH. Whether these proteins function together to control melanosome pH is unclear.

In summary, several questions concerning melanosome generation, trafficking, and pH balance exist. Existing zebrafish mutants, and zebrafish single or double mutants that can be quickly generated using ENU mutagenesis, can be used to understand the cell biology underlying aberrant pigmentation phenotypes. The ability to readily introduce additional HPS mutations using CRISPR and the high fecundity of zebrafish allows for large populations of genetically-modified zebrafish to be produced quickly, and it is advantageous for studying gene interactions in vivo [32,33,34,98]. Furthermore, the injected zebrafish embryos can then be grown into adults to start a new HPS model. Effects can be observed in injected embryos within days of injection. Although some mutations may ultimately be lethal, knowledge can be gained in mutants and CRISPR knockouts during larval stages, as performed in vacuolar protein sorting (Vps) 11 syndromic albinism models [29,99]. Furthermore, mutations can be introduced to specific regions of a gene in already existing HPS5 models such as *snow white,* which could provide valuable information on the function of specific protein domain interactions with existing mutations.

## 4. Diseases Involving Changes to Pigment Cell Development and Function

Changes to melanogenesis and to the growth and development of melanocytes/melanophores can cause disease. The mechanisms causing pigment diseases are varied. Some examples of pigment diseases and their mechanisms are: (1) melanoma resulting from the uncontrolled growth of melanocytes, and (2) albinism caused by altered melanogenesis. Certain diseases associated with pigmentation defects have systemic effects on the body due to the shared cellular machinery used during pigment trafficking and the excretion of cytotoxic granules in the immune system, neurotransmitters in the nervous system, and deposition of surfactants, which are a lipid secreted over alveola cells that help with alveolar expansion by lowering surface tension [13,100].

### 4.1. Melanoma

Melanoma is caused by the uncontrolled growth of melanocytes [101]. Melanoma cases are increasing in the United States, and if caught early enough, prognosis is good, but it declines with advancing stages [102,103]. In many ways, melanoma is a disease of pigment cell development. The processes that govern pigment cell development to specify, differentiate, and proliferate melanocytes are altered with deadly consequences in melanoma. For example, activation of the WNT pathway is important for the specification of melanoblasts, as it drives neural crests cells to a melanoblast fate instead of a neuron precursor fate [104]. Increased activation of the WNT/MITF pathways is associated with proliferative melanoma [105,106].

Melanoma can occur in the skin, on mucosal membranes, and in the eyes. The least understood melanomas are those of the mucosal membrane and eyes [101]. The mutational causes of these melanomas are less characterized, as opposed to melanoma of the skin. Mucosal melanomas tend to have increased copy numbers of genes critical for pigment development, such as MITF, which, when mutated, can make cancers resistant to treatment [107]. Furthermore, predictions of outcomes for patients requires a better understanding of the biomarkers that predict melanoma outcomes, which requires more research [101]. These biomarkers are genes that play a crucial role in normal pigment cell development such as MITF [101]. Therefore, understanding the development of pigment cells using cell models and animal models, such as zebrafish and mice, is crucial for developing treatments to melanoma (recently reviewed by Patton and colleagues [108]).

### 4.2. Albinism

Albinism is defined by the reduction or absence of melanin. Albinism can be broadly categorized into three types: (1) syndromic albinism due to mutations affecting lysosome-related organelles (LROs) and different systems in the body that rely on the proper biogenesis and function of LROs; (2) nonsyndromic albinism with symptoms confined to pigment loss and defects that arise from pigment loss; (3) albinism-associated disorders resulting from the loss of pigment-producing genes due to large chromosomal deletions, as is the case with Prader-Willi Syndrome and Angelman Syndrome. Only 1% of people with Prader-Willi Syndrome and Angelman Syndrome present with albinism, as their pigment-producing genes may escape deletion [109,110].

#### 4.2.1. Syndromic Albinism

The main forms of syndromic albinism are Chediak–Higashi Syndrome (CHS) and Hermanksy–Pudlak Syndrome (HPS). CHS is an extremely rare disease, as less than 500 cases have been reported. The disease is characterized by reduced amounts of neutrophils, natural killer cell defects, albinism, and platelet deficiency. Therefore, patients often die from infection when they are children, and treatments, such as a bone marrow transplant, are not always effective [111,112,113]. CHS is caused by loss-of-function mutations in the lysosomal trafficking regulator (indicated as LYST in human and as Lyst in mouse) [114,115]. Loss-of-function mutations in Lyst cause large, malformed melanosomes that are not evenly dispersed [116]. This effect is due to melanosomes fusing with each other, and LYST is likely involved in the correct localization of lysosome-related organelles and exocytosis, though its functions are still being determined [117,118,119].

Currently, there are 11 recognized types of Hermanksy–Pudlak Syndrome (HPS) [42]. HPS shares some symptoms with CHS because both diseases are the result of defective intracellular trafficking. Patients with HPS have seizures, show increased inflammation, increased risk of skin cancer, pulmonary fibrosis (lung scarring), varying degrees of albinism, visual problems, and increased mortality [120,121,122]. HPS is caused by mutations in the proteins of the BLOC complex or in proteins of the AP3 complex. Therefore, melanogenesis enzymes cannot reach the melanosome [120]. Additionally, the AP3 complex also traffics neurotransmitters, cytotoxic T-cell granules, and surfactant. HPS has a prevalence of 1/1,000,000 [13,123,124,125]. Current research is focused on reducing pulmonary fibrosis; however, there is no cure for HPS [125]. Mouse models for HPS exist, but there is a paucity of zebrafish models for HPS (Table 1). New zebrafish models are currently being characterized, and with quick genetic modification techniques, such as CRISPR, permanent specific gene loss-of-function models can be quickly developed to understand pigment gene function and protein/gene interactions in syndromic albinism.

#### 4.2.2. Nonsyndromic Albinism

Ocular albinism (OA), albinism of the eyes, and oculocutaneous albinism (OCA), albinism of the eyes and skin, are the main forms of nonsyndromic albinism. The diseases are characterized by a reduction in pigmentation, vision defects, and susceptibility to cancer [131]. These diseases are caused by mutations in the enzymes that create melanin (as is the case with OCA1 caused by mutations in TYR) or alter the melanosome environment required for proper melanogenesis enzyme function (as is the case with OCA2 caused by a mutation in the P-protein coded by OCA2) [131]. There are currently eight different types of oculocutaneous albinism and one type of ocular albinism [10]. OCA1 accounts for half of all albinism cases, while OCA2 accounts for 30% of cases, and zebrafish models exist for both of these conditions (Table 1) [21,132,133]. Patients with OCA1 and OA1 can be treated with a few drugs to stimulate melanogenesis [132]. The other diseases are managed by avoiding sun exposure, excision of cancerous lesions, and corrective lenses [45]. There is no cure for oculocutaneous albinism.

#### 4.2.3. Albinism Associated Disorders

Prader-Willi Syndrome (PWS) patients have sleep abnormalities, cognitive delay, abnormal differences in body structure, and obesity. PWS is caused by large chromosomal deletions on chromosome 15. The region that is deleted contains OCA2 and HERC2, two genes involved in pigmentation with HERC2 regulating the expression of OCA2 [134,135]. Without these genes, patients present with albinism along with PWS, but deletion of these genes is rare [109]. Angelman Syndrome (AS) patients present with developmental delay and seizures and such as PWS, results from deletions or changes in imprinting of chromosome 15. Albinism occurs from the deletion of OCA2, but albinism is not present in all cases [136].

## 5. Future Perspectives

### 5.1. The Genetics Underlying Pigmentation Disease Is Still Unfolding

It was found that, for 28% of patients with albinism, there were no mutations in known albinism-causing genes [137]. Therefore, we do not fully understand all the genes that cause albinism [138]. Indeed, new disease-causing mutations are being discovered as recently as 2020 [139,140]. Some mutations in pigment are first discovered in animal models before being described in humans, as is the case with the Mocha mouse, which was determined to have a defect in Ap3d1 in 1998 [141]. Hermanksy–Pudlak Syndrome type 10, which is caused by a mutation in AP3D1, was described in 2016, and it is rare [13,137,142]. While mouse models for HPS10 have been used to understand the role of the AP3 complex, there is a paucity of information on HPS10. Furthermore, by studying zebrafish albinism models that are not yet implicated in human albinism, we will gain a head start on clarifying the function of genes potentially underlying human albinism disorders. For example, the *fadeout* zebrafish mutation maps to chromosome 2 [143]. None of the genes currently implicated in human HPS have homologs on zebrafish chromosome 2. Therefore, if the *fadeout* gene was identified, we could screen people with suspected HPS for the mutation in the *fadeout* gene.

Because the different types of albinism can present in similar ways clinically, it is crucial to understand the genetics and cell biology of such patients [121]. Even conditions that, in theory, should be similar, may have slight differences. For example, HPS2 occurs when one subunit of the AP3 complex, AP3B1, is mutated. HPS10 is caused by mutations in AP3D1—another subunit of the AP3 complex. Patients with HPS2 tend to bleed excessively, which is not the case for patients with HPS10. Additionally, patients with HPS10 have severe neurological defects that are not characteristic of HPS2 [142]. Treatment strategies may differ for these patients, and correct diagnosis is crucial for alleviating symptoms. Furthermore, when studying each condition, one protein in a complex may function slightly differently than the other. It is best to have models for all proteins in a complex to obtain the clearest picture of their true function. Because zebrafish can be rapidly screened for gene function using CRISPR, they are excellent models for albinism gene discovery.

There is currently no cure for albinism at this time. However, there are possible therapeutic approaches for preventing albinism. While not available to everyone, in vitro fertilization (IVF) allows for the screening of embryos for genetic mutations that cause albinism before implantation is possible [144]. Knowing which genes and which mutations to screen for is therefore critical for this approach, and new gene editing technologies may allow for the treatment of these diseases before they develop.

With new genetic editing tools such as the CRISPR-Cas9 system, it is now possible to edit DNA in a specific manner. Theoretically, CRISPR-Cas9 could be used to edit out deleterious mutations in whole embryos. Alternatively, CRISPR-Cas9 could be used to repair mutations in melanocyte stem cells for implantation into the skin. Cell suspensions injected into the skin are capable of repigmenting skin in patients with vitiligo. This method requires taking cultured melanocytes from the patient and transplanting them elsewhere [145]. However, in patients with albinism without any pigmented skin, genetically-modified melanocytes derived from induced pluripotent stem cells or altered follicular melanocyte stem cells might offer an avenue for repigmentation [146]. A similar method has been used to correct mutations in alveolar cells from patients with HPS2 [147]. However, CRISPR modification comes with a risk of off-target effects, and the technology will have to be further developed to be used in gene therapy [148]. Zebrafish could be utilized to study these methods more in depth, as cells can be genetically modified in embryos, then transplanted into unedited mutant fish to test if genetic correction was successful.

### 5.2. The Role of Zebrafish Syndromic Albinism Models to Study and Find New Treatments for Pigment Disease

Certain albinism disorders are lethal to patients, as is the case with HPS10 [13,142]. The short lifespan of these patients means that there is very little time to better understand the disease to improve treatment options. Animal models provide the means to study disease progression and outcomes for rare diseases. For example, *crasher* homozygous recessive zebrafish carry mutations in *ap3d1*(hps10), and fish do not survive past two weeks of life (Neuffer et al., submitted). This is somewhat different than Mocha mice, with most surviving to adulthood even as homozygotes [141,149]. Furthermore, research in zebrafish may complement research in mice. For example, new albinism genes can be quickly discovered in zebrafish because of the rate at which gene knockouts can be performed in large numbers. Then, promising genes can be more closely studied in a mouse model. Additionally, biomarkers of disease outcomes may help monitor disease progression, but the available biomarkers to predict mortality in HPS are limited. Zebrafish models are better models to study premature death in patients with HPS, and they will be helpful in developing these biomarkers [120,150].

Additionally, albino zebrafish may also be used to study melanoma. At first glance, using an albino animal to study melanoma seems counterintuitive because people with albinism do not generate pigment, and therefore they would not generate pigmented lesions. However, if some pigment production is possible, then melanoma can result. In HPS1 caused by mutations in the biogenesis of lysosome-related organelle complex-3 (BLOC3) subunit 1, pigmented nevi (moles) are common and can develop into melanoma [122,151]. Previously, our lab utilized several zebrafish albinism models to understand chemotherapeutic resistance in melanoma [147]. These models and their corresponding albinism disorder were *mitfa* (Waardenburg Syndrome type 2), *vps11* (general syndromic albinism), and *oca2* (Oculocutaneous albinism 2) [19,29,152,153]. The chemotherapeutic drug, cisplatin, induces DNA damage and triggers apoptosis in melanoma cells [154]. However, melanoma cells can become resistant to cisplatin over time. A potential mechanism suggested by multiple groups underlying resistance include sequestration in mature melanosomes [155,156]. All of the albinism models we used to study resistance to cisplatin had defects in melanosome function, and melanophores from melanosome mutants were more sensitive to cisplatin-induced melanophore loss [153]. HPS zebrafish models have not been used to study chemoresistance. It could be expected that melanophores in current HPS zebrafish models could have increased sensitivity to cisplatin due to melanosome dysfunction, and therefore these zebrafish lines could serve as additional models for better understanding chemotherapeutic resistance in melanocytes.

## 6. Conclusions

The genetic similarity of melanogenesis and melanophore development genes, the availability of current zebrafish OCA and HPS models, and the ability to generate new ones through genetic engineering make zebrafish with albinism attractive models to understand not only albinism disorders but other skin pigmentation disorders.

## Figures and Tables

**Table 1:** Types of albinism disorders with some relevant mouse and zebrafish models.

Albinism Type	Human Disease	Human Gene	Protein	Mouse Model (Gene)	Zebrafish Model (Gene)
Syndromic	CHS	LYST	LYST	Beige Slate	*lyst^mu107^*
	HPS	RAB38	RAB38	Chocolate	Not available
	HPS1	HPS1	BLOC3, SUBUNIT 1	Pale ear	Not available
	HPS2	AP3B1	AP3BETA SUBUNIT 1	Pearl	Not available
	HPS3	HPS3	BLOC2, SUBUNIT 1	Cocoa	Not available
	HPS4	HPS4	BLOC3, SUBUNIT 2	Light ear	Not available
	HPS5	HPS5	BLOC2, SUBUNIT 2	Ruby-eye 2	*snow white (hps5)*
	HPS6	HPS6	BLOC2, SUBUNIT 3	Ruby-eye	Not available
	HPS7	DTNBP1	BLOC1, SUBUNIT 8	Sandy	Not available
	HPS8	BLOC1S3	BLOC1, SUBUNIT 3	Reduced pigmentation	Not available
	HPS9	BLOC1S6	BLOC1, SUBUNIT 6	Pallidin	Not available
	HPS10	AP3D1	AP3D1	Mocha	*crasher* (ap3d1 *)
	HPS11	BLOC1S5	BLOC1, SUBUNIT 5	Muted	
	HPS				*fadeout*
	HPS			Bloc1s1−/− [126]	*Bloc1s1^ihb815^* [127]
Nonsyndromic	OA1	GPR143		Oa-/y [128]	
	OCA1	TYR		Albino	*sandy* (*tyr*)
	OCA2	OCA2		Pink-eyed dilute	*oca2* [22]
	OCA3	TYRP1		Brown	Not available
	OCA4	SLC45A2		Underwhite	*albino (slc45a2)*
	OCA5	4q24		Not available	Not available
	OCA6	SLC24A5		Not available	*golden (slc24a5)*
	OCA7	C10ORF11		Not available	Not available
	OCA8	DCT		Slaty	Not available
Albinism-associated	PWS			PWS^ICdel^ [129]	Not available
	AS			Ube3a−/− [130]	Not available

* Neuffer et al., under revision.
